# The application of temperature sensitivity CRISPR/LbCpf1 (LbCas12a) mediated genome editing in allotetraploid cotton (*G. hirsutum*) and creation of nontransgenic, gossypol‐free cotton

**DOI:** 10.1111/pbi.13470

**Published:** 2020-09-14

**Authors:** Bo Li, Sijia Liang, Muna Alariqi, Fuqiu Wang, Guanying Wang, Qiongqiong Wang, Zhongping Xu, Lu Yu, Muhammad Naeem Zafar, Lin Sun, Huan Si, Daojun Yuan, Weifeng Guo, Yanqin Wang, Keith Lindsey, Xianlong Zhang, Shuangxia Jin

**Affiliations:** ^1^ National Key Laboratory of Crop Genetic Improvement Huazhong Agricultural University Wuhan Hubei China; ^2^ Xinjiang Key Laboratory of Crop Biotechnology Institute of Nuclear and Biological Technology Xinjiang Academy of Agricultural Sciences Urumqi Xinjiang China; ^3^ Xinjiang Production and Construction Corps Key Laboratory of Protection and Utilization of Biological Resources in Tarim Basin Tarim University Alaer Xinjiang China; ^4^ Department of Biosciences Durham University Durham UK

**Keywords:** genome editing, CRISPR, Cpf1, cotton, temperature sensitive, nontransgenic, glandless, gossypol‐free cotton

Cotton (*Gossypium hirsutum*) is an allotetraploid species and a typical thermophilic crop that can survive and grow well under temperatures up to 45 °C. CRISPR/LbCpf1 (LbCas12a) is a temperature‐sensitive system for plant genome editing (Malzahn *et al*., 2019) and has been successfully applied in species such as rice, soya bean, tobacco, maize and cotton (Lee *et al*., 2019; Li *et al*., 2018; Tang *et al*., 2017; Xu *et al*., 2019). However, the temperature sensitivity of LbCpf1 has not been tested in cotton yet, a high temperature‐resistant crop. In order to improve LbCpf1 efficiency and determine the optimum temperature for cotton genome editing, we investigated the effects of different temperatures on LbCpf1 activity and genome editing efficiency. Moreover, we created nontransgenic and glandless cotton plants with seeds free of gossypol, representing a valuable germplasm resource for cotton breeding.

In a development from our previous study (Li *et al*., 2019), we found that the observed albino phenotype of transgenic cotton plants containing LbCpf1‐*GhCLA*1 became enhanced and with an increase of temperature in plants grown in the field in Wuhan (Figure 1a). In order to determine the editing efficacy of LbCpf1 under elevated temperatures, 20 T1 seeds from a LbCpf1‐*GhCLA*1 transgenic line with whitish dappled leaves were sown in a growth chamber at either 24 °C, 29 °C, 34 °C or 37 °C for one week. We found that the cotyledons exhibited different degrees of albinism (Figure 1b, upper panel). All 20 plants showed dappled cotyledons at 24 °C. At 29 °C, 16 plants had some white spots and 4 plants had fully white cotyledons. At 34 °C, 13 plants were fully white, 3 plants were dappled and 4 plants had partially white cotyledons. In a parallel experiment, 20 T1 seeds were sown at 24 °C under the same conditions described above. When the plants developed the first true leaf, they were transferred to 24 °C, 29 °C, 34 °C or 37 °C for 2 weeks, with similar results as described for the previous experiment (Figure 1b, lower panel). However, long and continuous treatment at 37 °C significantly inhibited cotton growth, and some plants died. We determined the editing profiles of T1 seedlings using Sanger sequencing and High‐throughput sequencing (Hi‐Tom) (Liu *et al*., 2019). The results showed that each individual plant contained different editing frequencies, and the editing efficiency of *GhCLA*1 in both the At and Dt subgenomes was 68.4% at 24 °C, 85.3% at 29 °C and 89.7% at 34 °C (Figure 1c). The elevated editing efficiency (genotype) under the higher temperature was in accordance with the observed phenotypic changes under the various temperature regimes.

**Figure 1:**
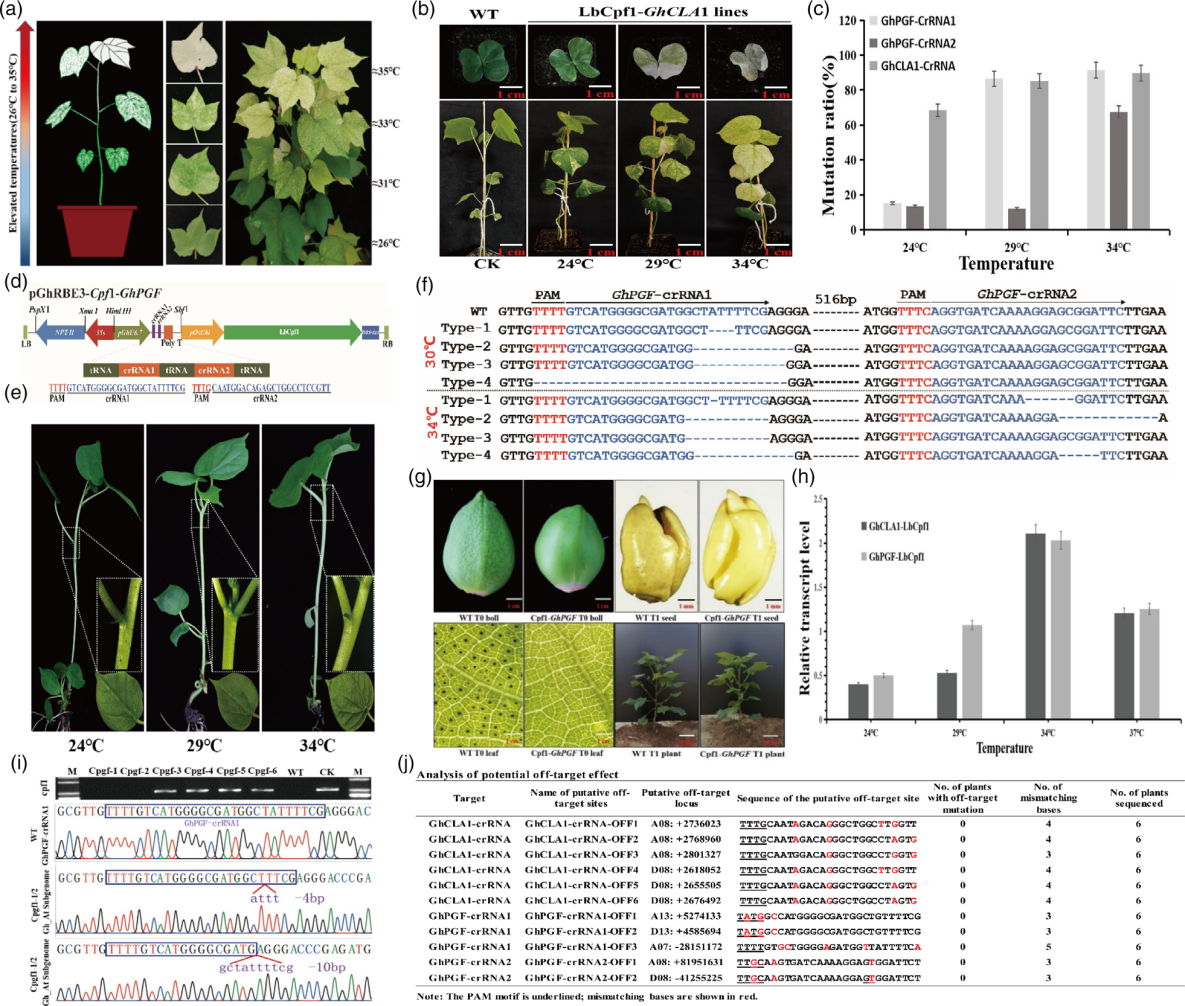
The application of CRISPR/ LbCpf1‐mediated genome editing in cotton and creation of gossypol‐free and transgene‐free cotton. (a) Development and phenotype of T1 plant with the target mutations in the *GhCLA*1 gene using the CRISPR/LbCpf1 system in the field under elevated temperatures in the summer of Wuhan City, China. With the increase of temperature, the bleaching phenotype of leaves becomes more obvious in T1 LbCpf1‐*GhCLA*1 transgenic plants grown in the field. (b) Phenotypes of T1 cotton plants with the target mutations in the *GhCLA*1 gene, grown at different temperatures. Upper panel: T1 seeds were sown at different temperatures (24 °C, 29 °C, 34 °C). The cotyledons of plants showed some leaf bleaching at 24 °C, whereas most plants were fully bleached at 34 °C. Lower panel: Plants were grown until emergence of the first true leaf at 24 °C and then transferred to different temperatures (24 °C, 29 °C, 34 °C) for two weeks, resulting in different degrees of bleaching. (c) Mutation frequencies in *GhPGF* and *GhCLA*1 genes in plants grown at different temperatures, revealed by Hi‐tom. The mutation frequency at the crRNA1 target sites is similar at 29 °C and 34 °C in LbCpf1‐GhCLA1 and LbCpf1‐GhPGF T0 plants; the editing efficiency at the crRNA2 target site was lower at 24 °C and 29 °C; LbCpf1 exhibited the highest efficiency at 34 °C. (d) T‐DNA region of the pGhRBE3‐Cpf1‐*GhPGF* vector, showing PAM and crRNAs sequences. The tRNA‐crRNA1‐tRNA‐crRNA2 transcription unit was constructed in the vector pGhRBE3‐Cpf1‐GhPGF. (e) Phenotype of T0 plants with target mutations in *GhPGF* under different temperature treatments. The glands in T0 plants were reduced at 24 °C and completely lacking at 29 °C and 34 °C. (f) Editing profile at *GhPGF* target sites in T0 plants. The editing types at the first crRNA target site are similar after different temperature treatments. The second crRNA target exhibits a lower editing efficiency at 24 °C and 29 °C, and both target sites showed editing at 34 °C. (g) Phenotype of *GhPGF* knockout mutants in T0 and T1 generations. Black dots in each picture show the glands on the surface of T0 cotton boll, leaf, T1 seed, seedling and wild‐type (WT) control. (h) Transcriptional activity of *LbCpf1* in cotton at different temperatures. Quantitative real‐time (qRT)‐PCR shows expression of *LbCpf1* in transgenic plants was highest at 34 °C. The internal reference gene was *GhUbi7* used here. (i) Identification of transgene‐free and glandless T1 cotton plants. Upper panel: The LbCpf1 sequences were detected in 4 plants but not in Cpgf‐1 and Cpgf‐2 plants; (m) DL2000 Marker; WT: Wild type; CK: Plasmid control. Lower panel: The edited *GhPGF* sequences in the two mutated plants (Cpgf‐1 and Cpgf‐2) showed a 4 bp indel (‐attt‐) and a 10 bp indel (‐gctattttcg‐). (j) Analysis of potential off‐target effect in LbCpf1‐*GhCLA*1 and LbCpf1‐*GhPGF* edited plants. Off‐target effects were detected at 11 predicted potential off‐target sites in 12 independent T0 plants by Sanger sequencing. The PAM motif is underlined; mismatching bases are shown in red.

Cotton seeds are a rich source of oil and protein, but are not edible due to the toxicity of the metabolite gossypol, which is stored in dark pigmented glands and is only produced in *Gossypium* species. Silencing the *PIGMENT GLAND FORMATION* (*PGF*) gene by RNAi leads to a glandless phenotype (Ma *et al*., 2016). To further test the efficiency of CRISPR/LbCpf1 system under different temperatures in cotton, we conducted a targeted editing in the coding sequence of *GhPGF* by using the tRNA‐crRNA1‐tRNA‐crRNA2 transcription unit developed in our recent report (Wang *et al*., 2018; Figure 1d).

LbCpf1‐GhPGF‐transformed somatic embyrogenic calli were grown in light incubators at 24 °C, 29 °C, 34 °C or 37 °C for one week. Thirty independent T0 plants were obtained from the treated calli. We found that glands were reduced in number in regenerated plants grown at 24 °C and were completely lacking at 29 °C and 34 °C (Figure 1e). Some calli grown at 37 °C showed abnormal development or death, and no plant data were obtained. Sanger sequencing results showed that the editing profiles at the first target were similar between plants grown under the different temperature treatments. The second target showed very low editing frequencies under 24 °C and 29 °C treatments, while at 34 °C, some editings were detected (Figure 1f). Hi‐tom sequencing results also showed that all 10 plants at 24 °C were edited at one *GhPGF* locus in both the At and Dt subgenomes. At 29 °C and 34 °C, 6/10 and 10/10 plants were mutated at four loci of *GhPGF*, with editing efficiencies at the GhPGF‐crRNA1 site of 15.2% (24 °C), 86.5% (29 °C) and 91.5% (34 °C), and at the GhPGF‐crRNA2 site, 13.6% (24 °C), 12.1% (29 °C) and 67.6% (34 °C) (Figure 1c). Correspondingly, both T0 and T1 plants produced glandless bolls, leaves and seeds (Figure 1g). Compared with the expression level at normal temperature (24 °C), transcription of *LbCpf1* in transgenic plants was found to be highest at 34 °C (Figure 1h). In order to obtain both glandless and transgene‐free plants, T1 plants were obtained by selfing T0 homozygous plants. The *LbCpf*1 gene was detected by PCR, and the target editing types of *GhPGF* gene were detected by Sanger sequencing. Two transgene‐free T1 plants with the target mutations (Cpgf‐1, Cpgf‐2) were obtained (Figure 1i), and both plants are glandless. Sequencing demonstrated that no off‐target mutations were detected in any predicted off‐target sites (Figure 1j).

The results presented demonstrate that 34 °C is the optimum temperature for active CRISPR/LbCpf1 in cotton, and the bleached, glandless phenotype facilitates analysis. More importantly, homozygous, nontransgenic and gossypol‐free plants provide valuable new germplasm for molecular breeding programs. The opportunity is demonstrated for improved editing efficiency in cotton by simple heat treatment during seed sowing or plant tissue culture.

## Author contributions

SX.J. and XL.Z. designed the project. B. L., S.J.L., F.Q.W., G.Y.W., QQ.W., ZP.X., L.S., L. Y., M.N.Z., H.S., DJ.Y., WF.G. and YQ.W. performed experiments and wrote the manuscript. SX.J., M.A. K.L. revised the manuscript.

## Conflicts of interest

The authors declare no competing financial interests.
